# Book Reviews

**Published:** 1799-03

**Authors:** 


### CRITICAL RETROSPECT
### OF
### *MEDICAL AND PHYSICAL LITERATURE.*

FOR FEBRUARY, 1799.

*** *This Department of our Journal will be continued regularly every Month. In the present Number, we are obliged, for want of room, to confine ourselves to the Review of the latest and most valuable foreign and domestic Publications.*

### NATURAL HISTORY AND BOTANY.

*Flora Atlantica, ſive hiſtoria plantarum, quæ in atlante, agro Tunetano et Algerienſi creſcunt; auctore* R. DESFONTAINES. Number firſt. Folio. *Paris.*

The firſt number of this ſplendid work contains the three claſſes of the Linnæan Syſtem, viz. the *Monandria, Diandria,* and *Triandria,* repreſented on thirty elegant engravings, and accompanied by the Latin text, or explanations, printed on fifteen ſeparate leaves, in a very ſuperior ſtyle.—We cannot withhold our praiſe and admiration from a work of ſuch magnitude, carried on in a country, and under circumſtances, the moſt unfavourable to expenſive and coſtly undertakings.

*Tableau élèmentaire,* &c. " *Elementary Picture of the Natural Hiſtory of Animals:*" by G. CUVIER, of the National Inſtitute of France, &c. 8vo. XVI. and 710 pp. with indexes, and fourteen copper-plates (8 liv.) *Paris.* Baudouin.

This valuable work is deſigned immediately for the uſe of the pupils ſtudying Natural Hiſtory in the central ſchools. It contains, however, ſuch a number of *new* obſervations on the prevailing ſyſtems of this ſcience, and ſo many conſiderable changes, that it is juſtly intitled to the attention of every naturaliſt.

*Auſwahl ſchöner Gewächſe,* &c. " *A Choice Collection of rare and beautiful plants: being a continuation of the American plants:*" Number III. from Table 201 to 250; fifty coloured plates, and one ſheet text; 8vo. (4 rix-dollars.) *Nürnberg.* Raſpe.

The engravings, as well as the colouring, is more correct and chaſte in this, than in the earlier numbers. Moſt of the plates are faithful copies from the works of our celebrated *Curtis,* and the no leſs famous botaniſt, Profeſſor *Jacquin,* of Vienna. From the magnificent collection of plants, by the latter, we find in this number ſeveral ſpecies of ſorrel (*Rumex acetoſa Lin.*) repreſented with truth and elegance.

*Nouvelle Mécanique, &c. A New Analyſis of the Mechaniſm of motion in Men and Animals:* by J. P. BARTHEZ, 8vo. *Paris.* Carcaſonne.

The author divides this treatiſe into ſix principal ſections, explaining ſucceſſively the mechaniſm of the different claſſes of animals; the progreſſive motions of man and quadrupedes, as well as of the animals that creep, ſwim, and fly.

In an introductory diſcourſe, written with dignity and perſpicuity, M. BARTHEZ ſtates his ideas reſpecting the nature of the vital principle, and the *firſt cauſe* of the phenomena he endeavours to explain.

